# *In silico*/computational analysis of mevalonate pyrophosphate decarboxylase gene families in *Campanulids*

**DOI:** 10.1515/biol-2021-0103

**Published:** 2021-09-22

**Authors:** Minghui Cui, Limei Lin, Hongyu Guo, Duoduo Zhang, Jie Zhang, Wenwen Cheng, Xin Song, Zhaobin Xing, Yuehong Long

**Affiliations:** College of Life Sciences, North China University of Science and Technology, Tangshan 063210, China

**Keywords:** *Campanulids*, Apiales, mevalonate pyrophosphate decarboxylase, gene family, adaptive gene evolution

## Abstract

Mevalonate pyrophosphate decarboxylase (*MPD*) is a key enzyme in terpenoid biosynthesis. *MPD* plays an important role in the upstream regulation of secondary plant metabolism. However, studies on the *MPD* gene are relatively very few despite its importance in plant metabolism. Currently, no systematic analysis has been conducted on the *MPD* gene in plants under the order Apiales, which comprises important medicinal plants such as *Panax ginseng* and *Panax notoginseng.* This study sought to explore the structural characteristics of the *MPD* gene and the effect of adaptive evolution on the gene by comparing and analyzing *MPD* gene sequences of different campanulids species. For that, phylogenetic and adaptive evolution analyses were carried out using sequences for 11 *Campanulids* species. *MPD* sequence characteristics of each species were then analyzed, and the collinearity analysis of the genes was performed. As a result, a total of 21 *MPD* proteins were identified in 11 *Campanulids* species through BLAST analysis. Phylogenetic analysis, physical and chemical properties prediction, gene family analysis, and gene structure prediction showed that the *MPD* gene has undergone purifying selection and exhibited highly conserved structure. Analysis of physicochemical properties further showed that the *MPD* protein was a hydrophilic protein without a transmembrane region. Moreover, collinearity analysis in Apiales showed that *MPD* gene on chromosome 2 of *D. carota* and chromosome 1 of *C. sativum* were collinear. The findings showed that *MPD* gene is highly conserved. This may be a common characteristic of all essential enzymes in the biosynthesis pathways of medicinal plants. Notably, *MPD* gene is significantly affected by environmental factors which subsequently modulate its expression. The current study’s findings provide a basis for follow-up studies on *MPD* gene and key enzymes in other medicinal plants.

## 1 Introduction

Mevalonate pyrophosphate decarboxylase (*MPD*) is an enzyme that belongs to the Galactokinase-Homoserine kinase (*GHMP*) superfamily. It plays a key role in the Mevalonate (MVA) pathway and is the least studied enzyme in the GHMP superfamily [1,2]. *MPD* catalyzes decarboxylation of hexafluorovalerate diphosphate to form isoprene pyrophosphate [3]. Its enzyme structure is highly conserved across different species. *MPD* comprises two identical subunits in yeast: a monomer and a fissure structure. Three reverse parallel β-folds separate the α-helix in the monomer, and the fissure structure is implicated in ATP binding and comprises conserved amino acid residues [4]. The gene that encodes *MPD* is ubiquitous in animals, plants, and microorganisms such as *Panax ginseng* [5], *Ganoderma lucidum* [6], *Bacopa monniera* [7], *Homo sapiens* [8], *Sus scrofa* [9], *Enterococcus faecalis* [10], and *Bifidobacterium bifidus* [11].

*MPD* is implicated in the synthesis of terpenoids in *Campanulids* plants. These compounds include triterpenoid saponins in *Panax ginseng* and carotene in *Daucus carota*. Genomes of these two species have been sequenced. MVA pathway is the main terpenoids synthesis pathway in plants [12]. Furthermore, the expression level of the *MPD* gene is positively correlated with the terpenoid synthesis rate in plants [13].

The genetic information of species changes and is modulated by natural selection as a biological evolution process to enable adaptation to the living environment. This process entails the adaptive evolution of genes [14]. Therefore, gene family and adaptive evolution analyses should be carried out and more species sequenced to explore their roles. The study of the *MPD* gene family is important as it is a key enzyme in the terpenoid biosynthesis pathway in medicinal plants. In the current study, *MPD* gene identification and adaptive evolution analyses of 11 species of *Campanulids* were performed on *P. ginseng* and its related species. *P. ginseng* is a medicinal plant and a member of the Araliaceae family [15]. *MPD* gene family analysis of *P. ginseng* and the related species provides valuable information for further study of the *MPD* gene family. The findings help elucidate existing gene variations, protein knot structural and functional changes, and the evolutionary history of the species [16]. The current study further provides a reference for the study of gene families of key enzymes in other medicinal plants.

## 2 Materials and methods

### 2.1 Sequence data

Sequencing data used in the current study were retrieved from two databases. Genome, protein, and annotation files of 11 *Campanulids* species were retrieved from the National Center for Biotechnology Information (NCBI) genome database. Another set of genome, protein, CDS, and annotation files was retrieved from the corresponding genome website of plants. Data for seven other plants with different genetic relationships were retrieved. BLASTp tool in NCBI was used to compare these data with the known *MPD* protein sequence of *Eleutherococcus senticosus*. *Arabidopsis thaliana* and *Hevea brasiliensis* sequences were selected as outgroups. The amino acid sequence of *MPD* was identified using the local BLAST tool and submitted to NCBI-Conserved Domain Database (CDD) for further screening. A total of 21 nucleotide sequences were obtained from the screening which were extracted from the genome by corresponding numbers using the FASTA extract function in TBtools [17]. Sequence data comprising 275–423 codons were obtained using the ClustalW program and the sequences were further aligned using Multalin software [18].

### 2.2 Construction of phylogenetic tree

MEGA7 software was used for analyzing the sequence characteristics and for evolutionary analysis. A phylogenetic tree was constructed using the maximum likelihood (ML) method with 1,000 bootstrap values. The trees were analyzed using ITOL (http://itol.embl.de) webserver.

### 2.3 Analysis of adaptive evolution

The phylogenetic tree file was first transformed into a Phylogenetic Analysis by Maximum Likelihood (PAML) file using the EasyCodeML software [19]. The branch-site model and site model in the codeML program of the PAML4.8 software package were used to analyze the phylogenetic trees [20]. Data were then submitted to Datamonkey (http://www.datamonkey.org/) [21] and MEC (http://selecton.tau.ac.il/) for analysis of adaptive evolution. The random-effects like (REL) model, fixed effects likelihood (FEL) model, and single likelihood ancestor counting (SLAC) were used to analyze the pressure of site selection using webservers. Positive selection using SLAC and FEL methods was set at a locus level of *P* < 0.1. A Bayesian factor of <50 was acceptable for REL method.

### 2.4 Prediction of basic physical and chemical properties, secondary structure, and three-dimensional structure of *MPD* protein

The *MPD* amino acid sequence identified by BLAST was submitted to the Swiss Institute of bioinformatics database (https://www.expasy.org/) to predict its basic physicochemical properties, secondary structure, and three-dimensional structure.

### 2.5 Motif analysis

Motifs present in the *MPD* amino acid sequences were analyzed using the MEME software (http://meme-suite.org). The parameters were set as follows: a total number of search motifs = 10, shortest motif length = 6, and the maximum motif length = 50. Results were visualized using the visualized meme/Master motif pattern function in TBtools.

### 2.6 Chromosome location analysis

Chromosome location information of the *MPD* gene in all species was found in the annotation files of related species in Apiales. Related species included *D. carota* and *Coriandrum sativum*. The chromosome location map of *MPD* gene was then drawn using mapchart software.

### 2.7 Collinearity analysis

Collinearity of *P. ginseng*, *D. carota,* and *C. sativum* was analyzed using the one step MCScanX function in TBtools. The results were presented using the Local Circos software.

### 2.8 Prediction of cis-acting elements and *MPD* gene structure

2,000 bp upstream nucleotide sequences of *MPD* genes in *P. ginseng*, *D. carota,* and *C. sativum* were extracted using the GTF/GFF3 sequence extractor function in TBtools. The sequences were submitted to the PlantCARE (http://bioinformatics.psb.ugent.be/webtools/plantcare/html/) web server for predictions of *cis*-acting elements [22]. The visualization gene structure-function in TBtools was used to predict *MPD* gene structure.

## 3 Results

### 3.1 Phylogenetic analysis

**Figure 1:**
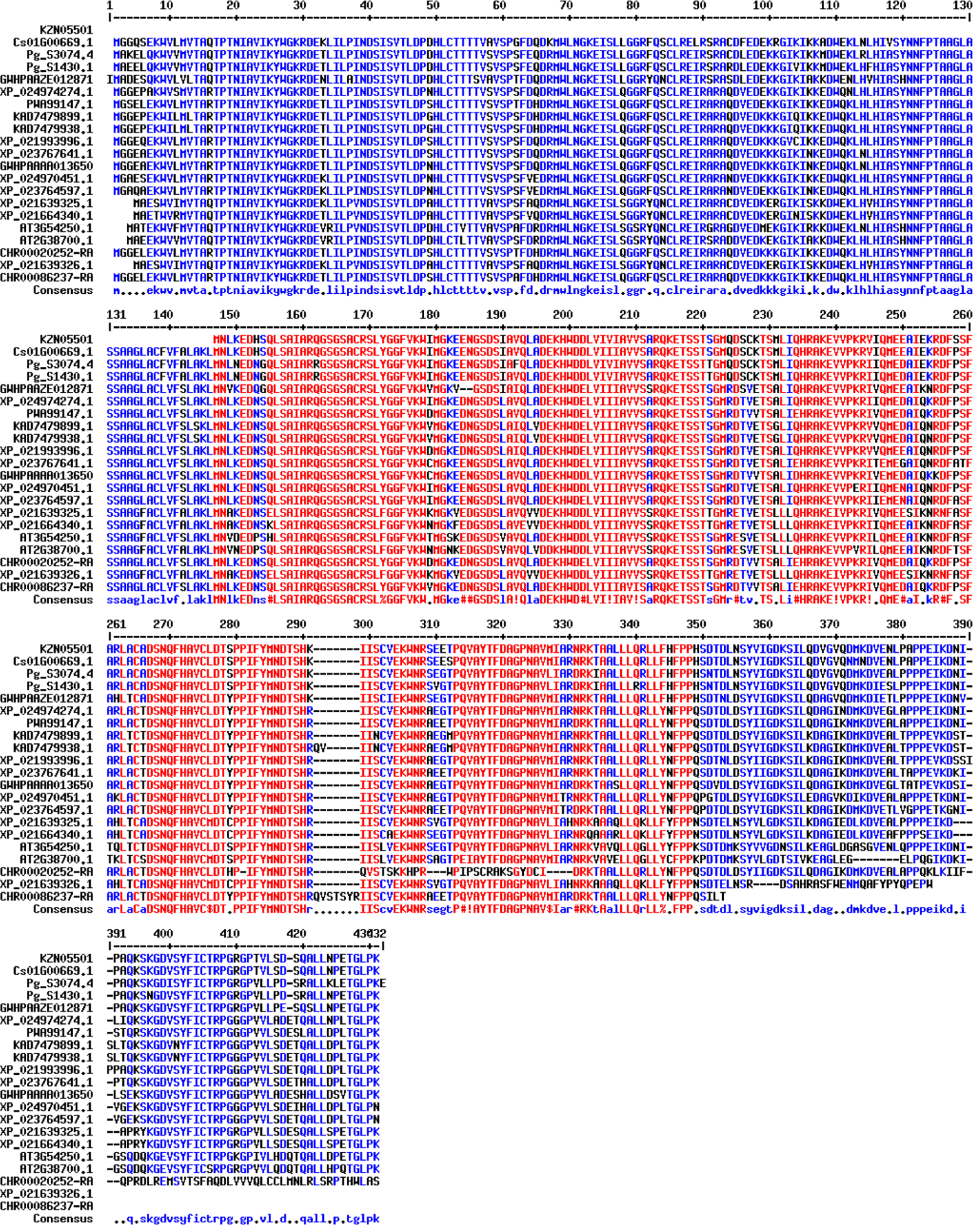
BLAST results of *Campanulids MPD* protein sequences. Amino acid sequence number is shown on the left, and the red sites indicate sites with high consensus. Blue sites represent low consensus and black sites represent neutral sites.

**Figure 2:**
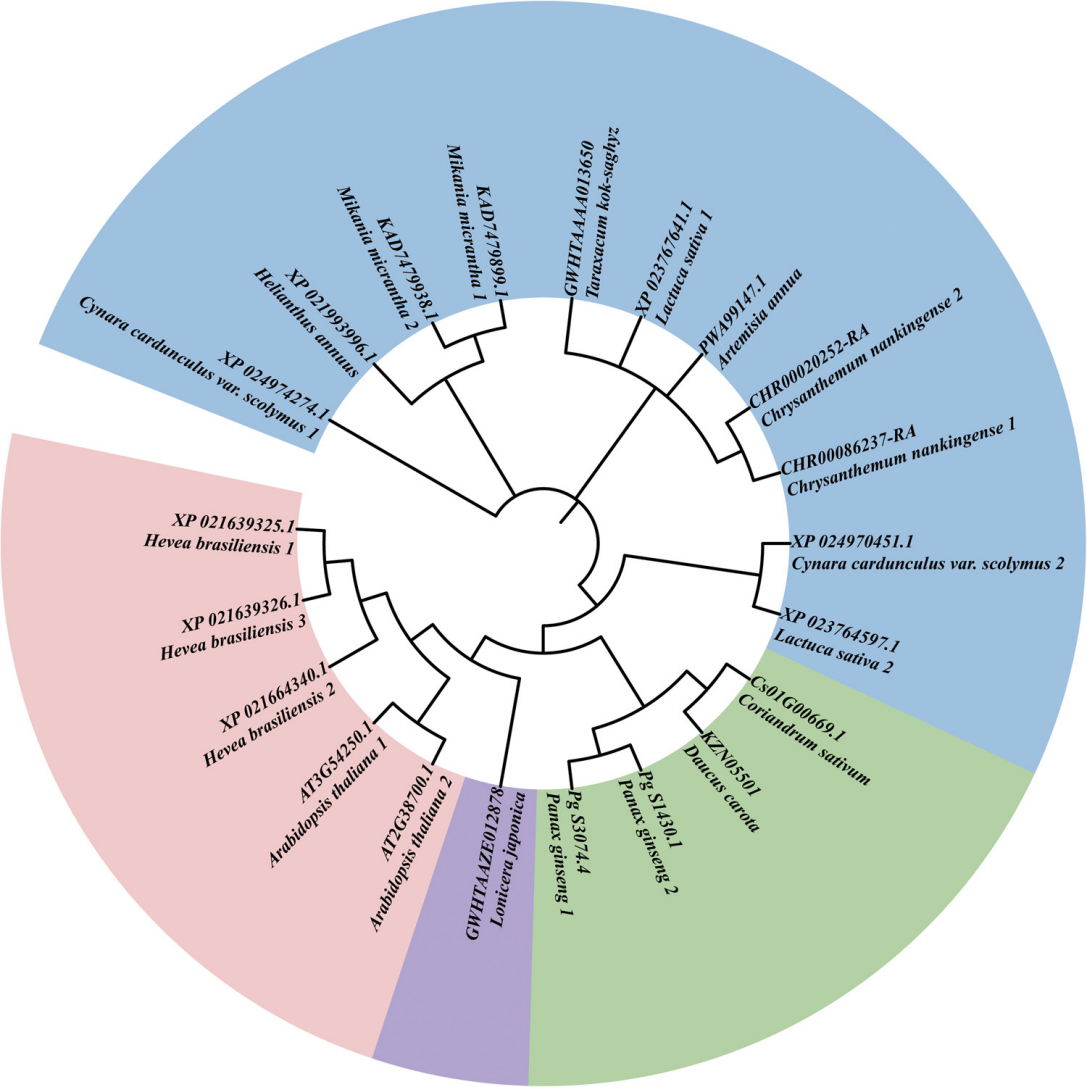
Phylogenetic tree based on the selected 21 *MPD* nucleotide sequences. The purple area shows Caprifoliaceae species, the green area indicates Araliaceae species, the blue area indicates Astraceae species, and the red area represents outgroups, based on ML method. Phylogenetic calculations were performed with 1,000 replicates.

BLAST results of 21 *MPD* sequences are shown in Figure 1. Sequence information and BLAST results of *Campanulids* are shown in Table 1. Sequence information and BLAST results of other plants are shown in Table S1. A phylogenetic tree was constructed based on the selected 21 *MPD* nucleotide sequences (Figure 2). A phylogenetic tree based on *MPD* protein sequences of all downloaded species is shown in Figure S1. *Lonicera japonica* was isolated as a Caprifoliaceae. *Cynara cardunculus* var. *scolymus*, *Lactuca sativa*, *Mikania micrantha*, *Helianthus annuus*, *Taraxacum kok saghyz*, *Artemisia annua*, and *Chrysanthemum nankingense* were grouped into Asteraceae, whereas *D. carota* and *C. sativum* clustered into Apiaceae. *P. ginseng* was isolated as an Araliaceae, and clustered with Apiaceae of Apiales. All the inner group species belonged to the *Campanulids* species. The outgroups (*A. thaliana* and *H. brasiliensis)* clustered into one branch (Figure 2). *Campanulids* clustered together, and branched into Asterales and Apiales (Figure S1). Other species with different genetic relationships clustered into one branch. *A. thaliana* and *H. brasiliensis* clustered into one branch, and *Zea mays* and *Amborella trichopoda* clustered into one branch. Further analysis showed that *Campanulids* have better clustering effect and higher sequence similarity, approximately 94.52%, whereas other species with distant genetic relationship showed lower *MPD* sequence similarity, approximately 82.19% (Table 1 and Table S1). These findings were consistent with the findings on the traditional taxonomy.

**Table 1:** Details on *Campanulids MPD* sequences

Sequences number	Species	Sources	Max score	Total score	Query cover (%)	Per. identity (%)
KZN05501	*Daucus carota*	http://apiaceae.njau.edu.cn:8080/carrotdb/	531	531	65	91.64
Cs01G00669.1	*Coriandrum sativum*	http://cgdb.bio2db.com/	766	766	100	89.52
Pg_S3074.4	*Panax ginseng*	http://ginsengdb.snu.ac.kr/	800	800	100	94.52
Pg_S1430.1	*Panax ginseng*	http://ginsengdb.snu.ac.kr/	785	785	100	94.05
GWHPAAZE012871	*Lonicera japonica*	https://bigd.big.ac.cn/gwh/Genome/98/show	736	736	100	85.24
XP_024974274.1	*Cynara cardunculus* var. *scolymus*	NCBI	698	698	100	84.32
XP_024970451.1	*Cynara cardunculus* var. *scolymus*	NCBI	692	692	100	82.86
XP_023767641.1	*Lactuca sativa*	https://lgr.genomecenter.ucdavis.edu/	704	704	100	81.47
XP_023764597.1	*Lactuca sativa*	https://lgr.genomecenter.ucdavis.edu/	701	701	100	81.67
KAD7479899.1	*Mikania micrantha*	NCBI	701	701	100	80.81
KAD7479938.1	*Mikania micrantha*	NCBI	697	697	100	80.42
XP_021993996.1	*Helianthus annuus*	NCBI	709	709	100	82.03
GWHPAAAA013650	*Taraxacum kok-saghyz*	https://bigd.big.ac.cn/gwh/Genome/1/show	701	701	100	81.47
PWA99147.1	*Artemisia annua*	NCBI	673	673	100	83.14
CHR00086237-RA	*Chrysanthemum nankingense*	http://www.amwayabrc.com/download.htm	579	579	81	83.95
CHR00020252-RA	*Chrysanthemum nankingense*	http://www.amwayabrc.com/download.htm	536	536	90	77.25

### 3.2 Identification and analysis of positive selection sites

Selection pressure of each locus in the MPD family was determined using the codeML tool in PAML software. The results are shown in Tables 2–4. Parameters for single rate model M0 and discrete model M3 were np = 30 and InL = −3079.912578, respectively. Parameters for discrete model M3 were np = 34 and InL = −3035.117992. LRT test between them was *P* < 0.01. The alternative hypothesis model M3 was upheld as true because M3 was significantly better compared with M0. This finding indicated that there were differences in selection pressure among different loci. The findings showed that *ω*_1_ and *ω*_2_ of M3 were less than 1 and there was no positive selection site. The LRT test value of M1a and M2a was *P* = 1 indicating that the alternative hypothesis M2a was not true. The LRT test value for M7 and M8 was *P* > 0.01 indicating that M8 model was not valid.

**Table 2:** *MPD* gene adaptive evolution analysis results based on site model with PAML

Model	np	Estimate of parameters	InL	LRT pairs	df	2ΔInL	*P*	Positively selected sites
M0: One ratio	31	*ω* = 0.08236	−3079.471949					
M3: Discrete	35	*P*_0_ = 0.57648, *P*_1_ = 0.39055, *P*_*2*_ = 0.03297	−3034.641386	M0/M3	4	89.66113	*P* < 0.01000	None
		*ω*_0_ = 0.00000, *ω*_1_ = 0.18815, *ω*_2_ = 0.56652						
M1a: Neutral	32	*P*_0_ = 0.93615, *P*_1_ = 0.06293	−3063.509873					Not allowed
		*ω*_0_ = 0.06385, *ω*_1_ = 1.00000						
M2a: Selection	34	*P*_0_ = 0.93615, *P*_1_ = 0.04464, *P*_2_ = 0.01922	−3063.509873	M1a/M2a	2	0	1.00000	None
		*ω*_0_ = 0.06295, *ω*_1_ = 1.00000, *ω*_2_ = 1.00000						
M7: Beta	32	*P* = 0.28411, *q* = 2.69822	−3036.445058					
M8: Beta＆*ω*	34	*P*_0_ = 0.99999, *P* = 0.28413, *q* = 2.69863	−3036.445320	M7/M8	2	0.00052	*P* > 0.10000	Not allowed
		*P*_1_ = 0.00001, *ω* = 1.00000						

Selection analysis by site models was performed using codeML implemented in PAML. np: number of free parameters. InL: loglikelihood. LTR: likelihood ratio test. df: degrees of freedom. 2ΔInL: twice the log-likelihood difference of the models compared.

**Table 3:** *MPD* gene adaptive evolution analysis results based on branch model with PAML

Models	np	Estimate of parameters	InL	LRT pairs	Df	2ΔInL	*P*
Branch A	32	*ω*_0_ = 0.08302	−3079.378725	M0＆MA	1	0.18645	0.00000
Branch B	32	*ω*_0_ = 0.08349	−3079.652431	M0＆MB	1	0.36096	0.00000
MF	57	*ω*_A_ = 0.544485	−3034.840869	M0＆MF	26	86.94158	1.71541 × 10^−8^
		*ω*_B_ = 0.0001					

**Table 4:** *MPD* gene adaptive evolution analysis results based on branch-site model with PAML

Models	np	Estimate of parameters	InL	LRT pairs	df	2ΔInL	*P*
Ma	34	*P*_2a_ = 0.00000, *P*_2b_ = 0.00000	−3063.509873				
		*ω*_b1_ = 1.00000, *ω*_b2b_ = 1.00000					
		*ω*_f_ = 1.00000, *ω*_0_ = 0.06293					
Ma_0_	33	*P*_2a_ = 0.00000, *P*_2b_ = 0.00000	−3063.509873	Ma_0_＆Ma	1	0.00000	1.00000
		*ω*_b1_ = 1.00000, *ω*_b2b_ = 1.00000					
		*ω*_f_ = 1.00000, *ω*_0_ = 0.06293					
Mb	34	*P*_2a_ = 0.00000, *P*_2b_ = 0.00000	−3063.509873				
		*ω*_b1_ = 1.00000, *ω*_b2b_ = 1.00000					
		*ω*_f_ = 1.00000, *ω*_0_ = 0.06293					
Mb_0_	33	*P*_2a_ = 0.00000, *P*_2b_ = 0.00000	−3063.509873	Mb_0_＆Mb	1	0.00000	1.00000
		*ω*_b1_ = 1.00000, *ω*_b2b_ = 1.00000					
		*ω*_f_ = 1.00000, *ω*_0_ = 0.06293					

SLAC model, IFE model, and REL model were used to identify positive selection sites based on Datamonkey test selection pressure. SLAC model detected 1 positive selection site and 95 negative selection sites when *P* was <0.1, and 0 positive selection sites when *P* was <0.01. A total of 21 negative selection sites were identified. Analysis using IFEL model identified six positive selection sites (48S, 93q, 98K, 178d, 226v, and 419A) and 167 negative selection sites when *P* was <0.1. With *P* <0.01, 0 positive selection sites and 51 negative selection sites were identified. REL detection was statistically significant when the significance level was 50. No positive selection sites were identified using REL model. However, 192 negative selection sites were detected.

**Figure 3:**
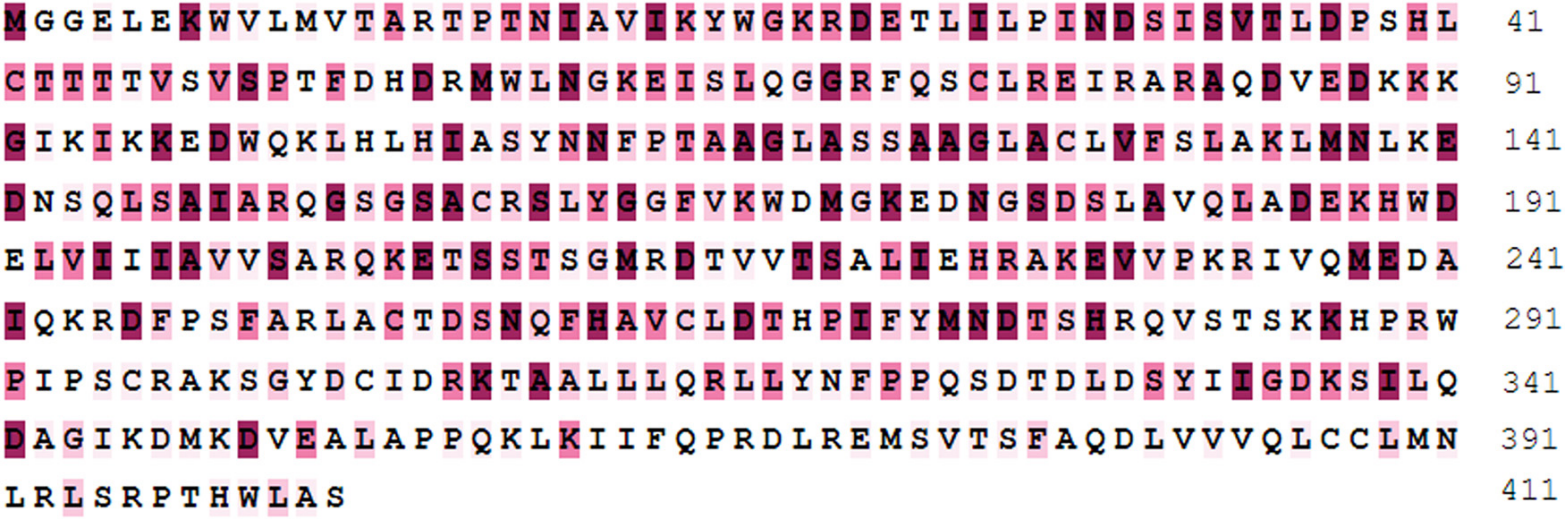
*MPD* gene analysis results based on MEC model. The neutral site is shown in white, the site of purification selection is shown in white to purple, and the statistically significant negative selection site is shown in dark purple color.

The 21 *MPD* CDS sequences were uploaded to MEC webserver for analysis using MUSCLE tool. Most of the loci were marked in purple (Figure 3). A total of 83 dark purple sites were identified which were strong negative selection sites. These sites accounted for 20% of the total sites. Analysis did not show positive selection sites marked in orange and yellow (Figure 3). This indicated that purified selection played a dominant role in evolution of the *MPD* gene family. This result was consistent with the findings from PAML and Datamonkey that negative selection was dominant in adaptive evolution of the *MPD* gene family.

### 3.3 Three-dimensional structure prediction of the *MPD* gene

**Figure 4:**
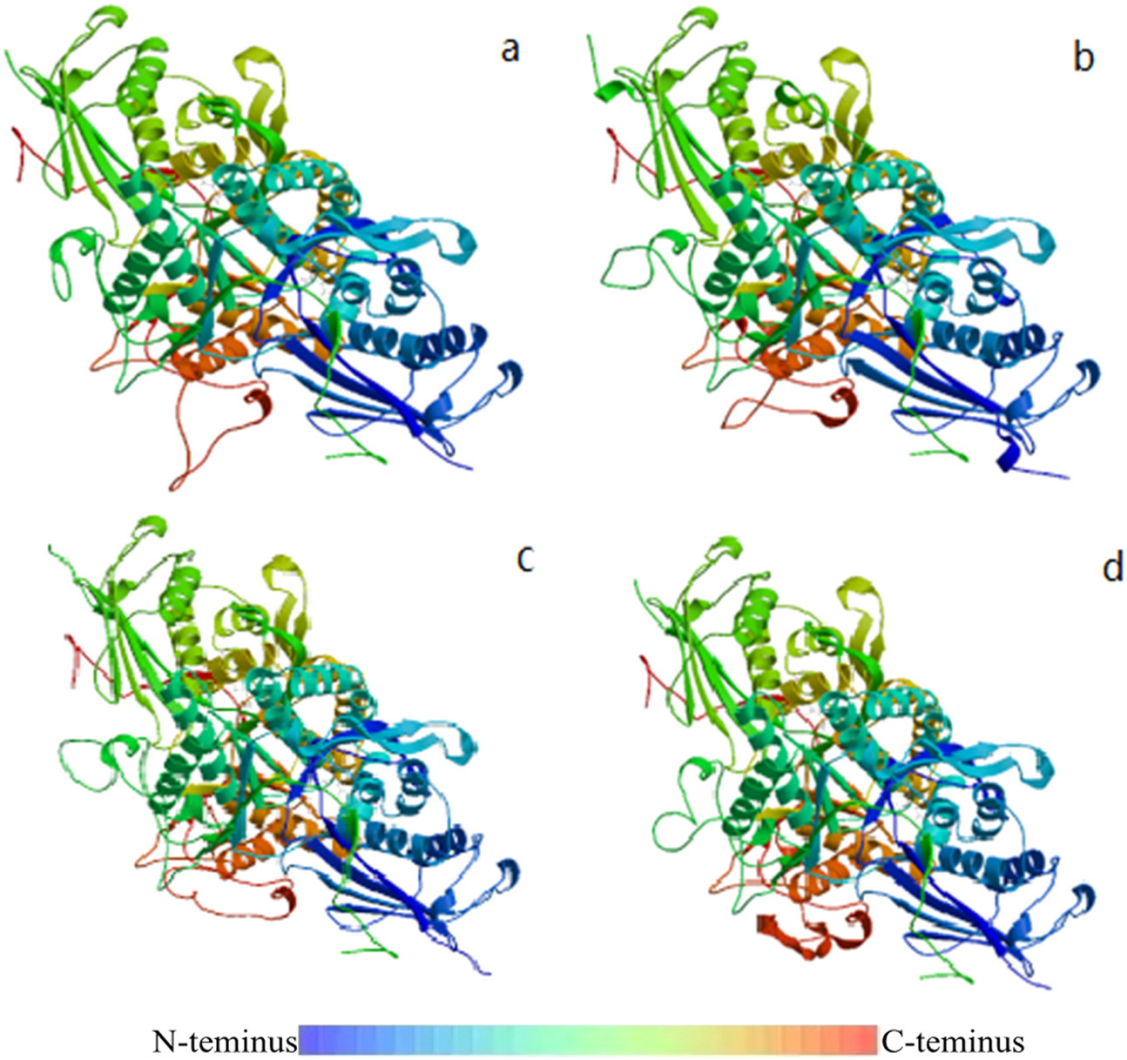
Prediction results of three-dimensional structure of *MPD* protein in related species of Apiales. (a) The three-dimensional structure of *C. sativum MPD* protein; (b) and (c) are the three-dimensional structure of the two *MPD* proteins in *P. ginseng*; (d) Three-dimensional structure of *E. senticosus MPD* protein with known sequence.

Physicochemical properties of all identified *MPD* amino acid sequences are shown in Table 5. The average length of *MPD* amino acid sequences was 420 amino acids. The relative molecular weight ranged between 45,586.00 and 919.55, whereas the isoelectric point ranged between 5.89 and 8.58. The hydrophilic range was between −0.207 and −0.308 except for *D. carota* (KZN05501), *Chrysanthemum nankingense* (CHR00086237-RA), and *H. brasiliensis* (XPXP_021639326.1). All the proteins were hydrophilic and had no transmembrane region. Predicted results of the protein secondary structures of identified *MPD* amino acid sequences are shown in Table 6. *MPD* amino acid sequences of *P. ginseng* and *C. sativum* of other Apiales plants were selected for modeling because the length of the *MPD* amino acid sequence of *D. carota* was too short to be used for comparisons. The *MPD* amino acid sequence of *E. senticosus* (AFM77982.1) with a known sequence and structure was used as the template sequence in the model. Modeling results are shown in Figure 4 and Table S2. The proportions of α-helix, β-turn, irregular coil, and extended chain are shown in Table 6. The proportions ranged between 35 and 40%, 4 and 5.5%, 35 and 40%, and 17 and 19%, respectively. Findings from BLAST analysis showed that similarity of all sequences was more than 75%. Most sequences had a similarity more than 80%. Three-dimensional structure prediction showed that the four *MPD* protein sequences were homodimers, and the ligands were 2 × DP6: (3*R*)-3-hydroxy-5-{[(*R*)-hydroxy (photonooxy) phophoryl] oxy}-3-methylpentanoic acid. Structure validations showed that GMQE values of the structures were 0.88–0.89, and QMEAN values were −1.79 to −1.34. The sequences of diphosphonate decarboxylase were matched in SWISS-MODEL, and the similarity was 74.63–76.72%, This indicates that spatial structure of *MPD* proteins has high similarity.

**Table 5:** Prediction results of basic physicochemical properties of identified *MPD* amino acid sequences

Sequences number	Amino number	Relative molecular mass	Isoelectric point (PI)	Hydrophilicity/average value of hydrophobicity	Transmembrane area
KZN05501	275	30424.53	5.99	Hydrophilic protein/−0.373	None
Cs01G00669.1	420	46449.06	6.52	Hydrophilic protein/−0.275	None
Pg_S3074.4	421	46785.80	7.54	Hydrophilic protein/−0.235	None
Pg_S1430.1	420	46618.45	6.76	Hydrophilic protein/−0.221	None
GWHPAAZE012871	419	46270.81	6.45	Hydrophilic protein/−0.255	None
GWHPAAAA013650	421	46256.67	5.89	Hydrophilic protein/−0.246	None
CHR00086237-RA	352	39283.00	8.58	Hydrophilic protein/−0.231	None
CHR00020252-RA	412	46118.99	8.58	Hydrophilic protein/−0.274	None
PWA99147.1	421	46607.16	5.97	Hydrophilic protein/−0.266	None
XP_021993996.1	423	46639.18	6.41	Hydrophilic protein/−0.297	None
KAD7479899.1	422	46692.29	6.47	Hydrophilic protein/−0.308	None
KAD7479938.1	424	46919.55	6.47	Hydrophilic protein/−0.305	None
XP_023767641.1	421	46448.93	6.05	Hydrophilic protein/−0.287	None
XP_023764597.1	421	46481.99	5.90	Hydrophilic protein/−0.264	None
XP_024974274.1	421	46429.96	6.21	Hydrophilic protein/−0.265	None
XP_024970451.1	421	46394.94	6.12	Hydrophilic protein/−0.254	None
XP_021639325.1	415	45780.37	6.76	Hydrophilic protein/−0.207	None
XP_021664340.1	415	45898.27	6.28	Hydrophilic protein/−0.236	None
XP_021639326.1	369	41213.97	7.60	Hydrophilic protein/−0.244	None
AT3G54250.1	419	46247.55	6.03	Hydrophilic protein/−0.293	None
AT2G38700.1	412	45586.00	6.33	Hydrophilic protein/−0.277	None

**Table 6:** Prediction results of secondary structure of identified *MPD* amino acid sequences

Sequences number	α-helix (%)	β-turn(%)	Random coil (%)	Extended strand (%)
KZN05501	40.00	4.73	40.36	14.91
Cs01G00669.1	35.71	4.05	42.14	18.10
Pg_S3074.4	38.95	4.04	38.72	18.29
Pg_S1430.1	36.19	3.33	43.33	17.14
GWHPAAZE012871	37.47	4.77	39.38	18.38
GWHPAAAA013650	38.00	5.23	39.19	17.58
CHR00086237-RA	39.20	5.40	36.93	18.47
CHR00020252-RA	42.23	4.13	35.44	18.20
PWA99147.1	33.73	4.75	42.76	18.76
XP_021993996.1	37.35	4.73	40.19	17.73
KAD7479899.1	36.49	5.45	40.52	17.54
KAD7479938.1	40.80	4.95	37.03	17.22
XP_023767641.1	38.24	5.23	38.00	18.53
XP_023764597.1	39.67	4.51	38.00	17.81
XP_024974274.1	39.19	4.28	39.43	17.10
XP_024970451.1	34.68	4.99	43.23	17.10
XP_021639325.1	38.07	5.30	38.07	18.55
XP_021664340.1	37.59	4.10	39.04	19.28
XP_021639326.1	42.55	2.71	37.94	16.80
AT3G54250.1	39.62	4.06	37.47	18.85
AT2G38700.1	39.81	5.10	36.89	18.20

### 3.4 Motif analysis

**Figure 5:**
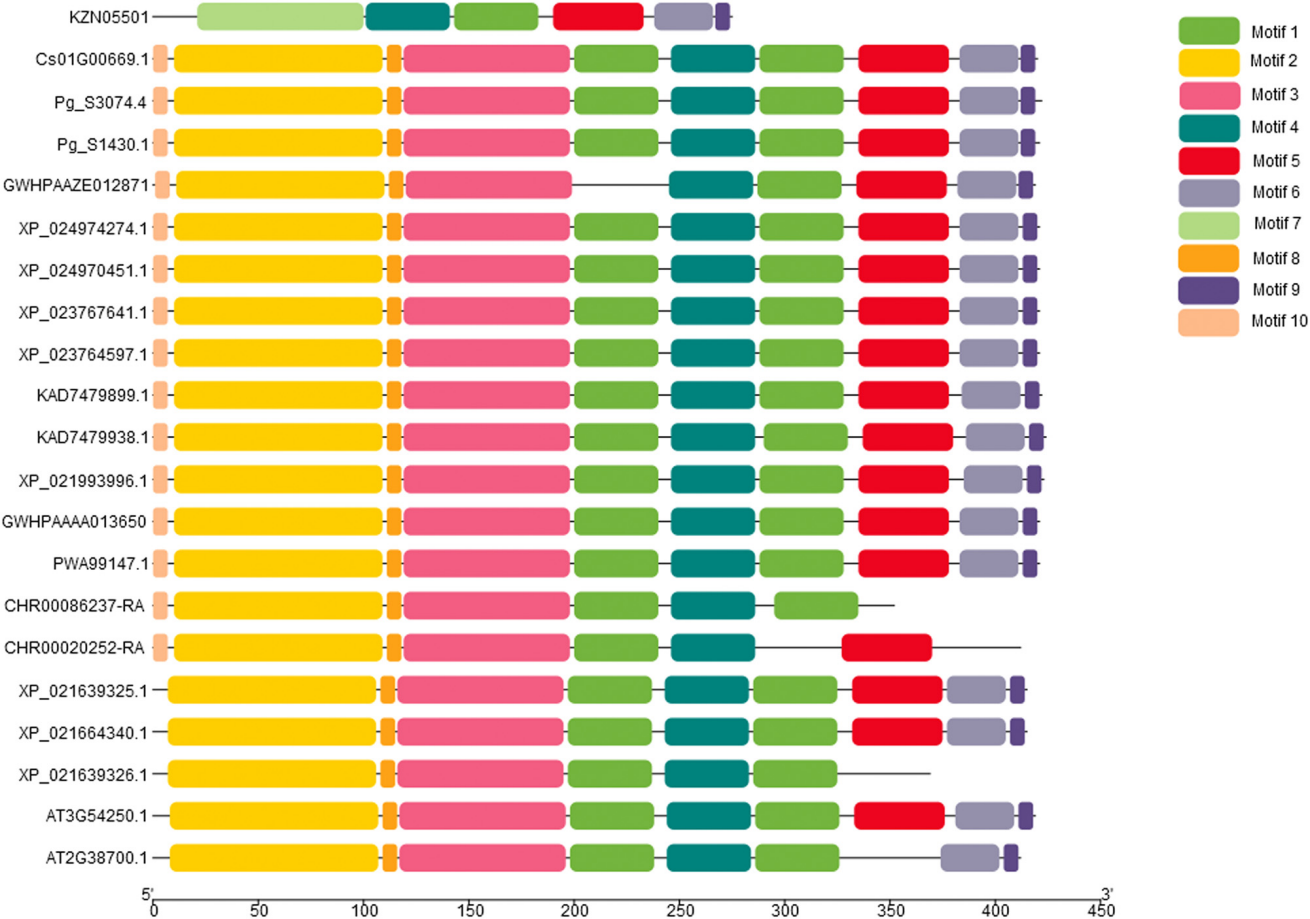
Motif analysis results of *MPD* amino.

Structural differences of *MPD* amino acid sequences were subtle. Most of the motif structures were highly similar with only a few having significant differences (Figure 5). Motif 1 and Motif 4 were present in all sequences, whereas Motif 7 was present only in the *MPD* protein sequence of *D. carota* (KZN05501). The first motif of the *MPD* protein sequence of *D. Carota* (KZN05501) was deleted owing to the short length of the sequence (275 amino acids). The other motifs were highly similar to those of other sequences. Motif 1 at the middle was not present in *L. japonica* (GWHPAAZE012871). In addition, the first sequence of *Chrysanthemum nankingense* (CHR00086237-RA) was shorter compared with that of the third sequence of *H. brasiliensis* (XP_021639325.1) (amino acids at 352 and 369), thus Motif 5, Motif 6, and Motif 9 at the end of the second sequence of *C. nankingense* (CHR00020252-RA) were deleted. Motif 10 was not present in three sequences of *H. brasiliensis* (XP_021639325.1, XP_021664340.1, and XP_021639326.1) and two sequences of *A. thaliana* (AT3G54250.1 and AT2G38700.1) amongst all the inner taxa.

### 3.5 Chromosome location analysis

**Figure 6:**
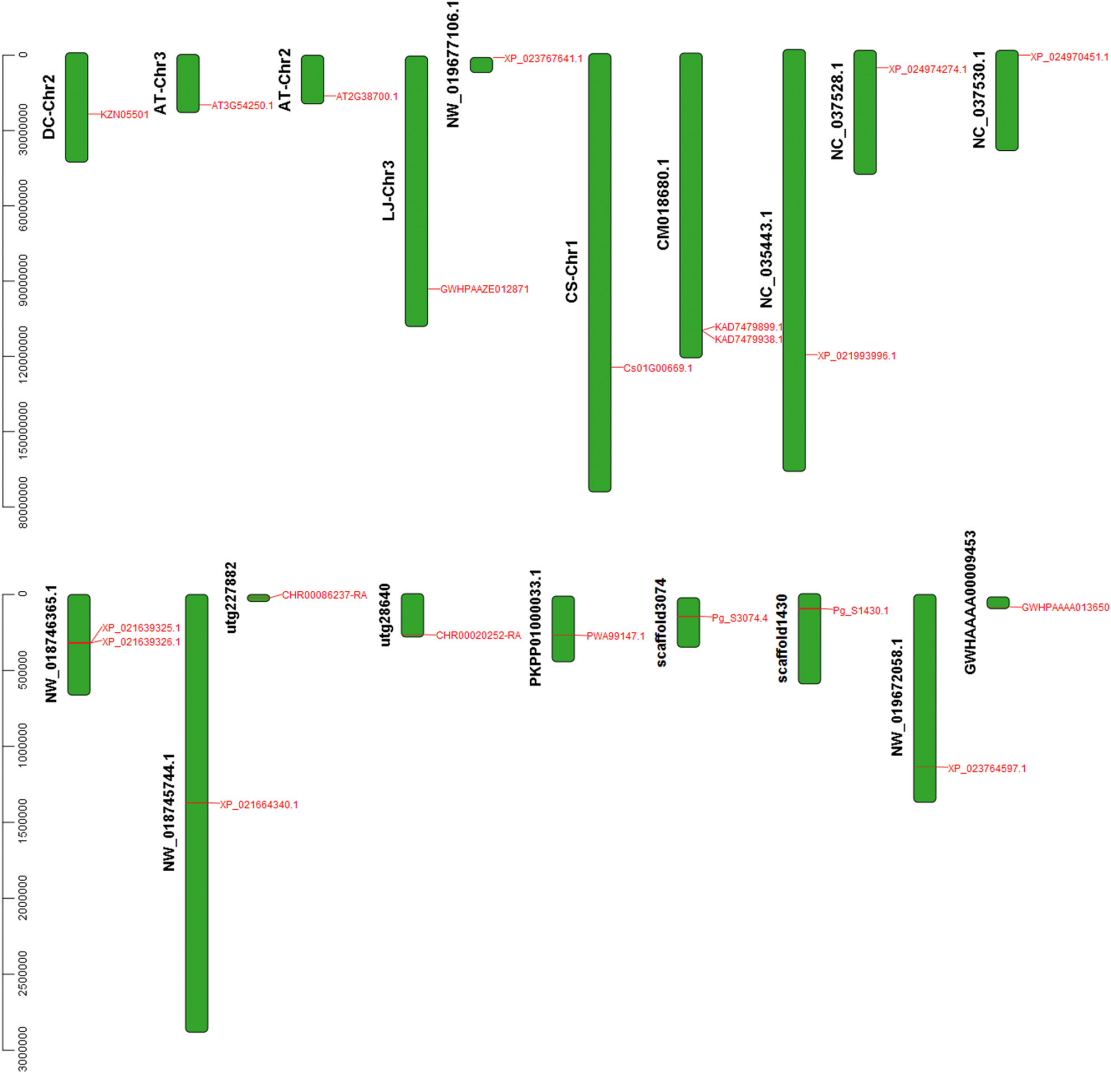
Location map of *MPD* gene in chromosomes and scaffolds.

The chromosome/scaffold length and *MPD* gene location of each species were obtained using chromosome/scaffold annotation files of each species. The results were then analyzed using TBtools (Figure 6). Notably, only *D. carota*, *C. sativum*, *L. japonica,* and *A. thaliana* had complete chromosome assembly information among the different species. The other species were mapped based on the scaffold level. The number of chromosomes, scaffolds, sequences, and species corresponding information are shown in Table 7.

**Table 7:** Corresponding table of chromosomes or scaffolds and sequences number

Chromosomes and scaffolds number	Sequences number	Species
DC-Chr2	KZN05501	*Daucus carota*
AT-Chr3	AT3G54250.1	*Arabidopsis thaliana*
AT-Chr2	AT2G38700.1	*Arabidopsis thaliana*
LJ-Chr3	GWHPAAZE012871	*Lonicera japonica*
NW_019677106.1	XP_023767641.1	*Lactuca sativa*
CS-Chr1	Cs01G00669.1	*Coriandrum sativum*
CM018680.1	KAD7479899.1	*Mikania micrantha*
CM018680.1	KAD7479938.1	*Mikania micrantha*
NC_035443.1	XP_021993996.1	*Helianthus annuus*
NC_037528.1	XP_024974274.1	*Cynara cardunculus* var. *scolymus*
NC_037530.1	XP_024970451.1	*Cynara cardunculus* var. *scolymus*
NW_018746365.1	XP_021639325.1	*Hevea brasiliensis*
NW_018746365.1	XP_021639326.1	*Hevea brasiliensis*
NW_018745744.1	XP_021664340.1	*Hevea brasiliensis*
utg227882	CHR00086237-RA	*Chrysanthemum nankingense*
utg28640	CHR00020252-RA	*Chrysanthemum nankingense*
PKPP01000033.1	PWA99147.1	*Artemisia annua*
scaffold3074	Pg_S3074.4	*Panax ginseng*
scaffold1430	Pg_S1430.1	*Panax ginseng*
NW_019672058.1	XP_023764597.1	*Lactuca sativa*
GWHAAAA00009453	GWHPAAAA013650	*Taraxacum kok-saghyz*

### 3.6 Collinearity analysis

**Figure 7:**
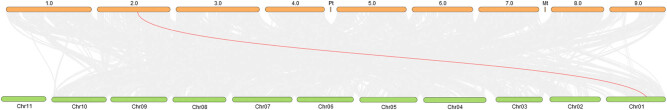
Collinearity analysis results between *D. carota* and *C. sativum* genomes. The yellow part indicates the completed chromosomes of *D. carota*, the green part indicates the completed chromosomes of *C. sativum*. The gray line indicates all collinearity analysis between the two genomes, and the red line indicates the existence of collinear *MPD* gene pairs.

MCScanX tool was used to analyze the genomes of *D. carota* and *C. sativum* to identify any collinearity. The findings showed that *MPD* gene of *D. carota* chromosome 2 (KZN05501) was collinear with that of *C. sativum* chromosome 1 (Cs01G00669.1) (Figure 7).

### 3.7 Prediction of cis-acting elements

**Figure 8:**
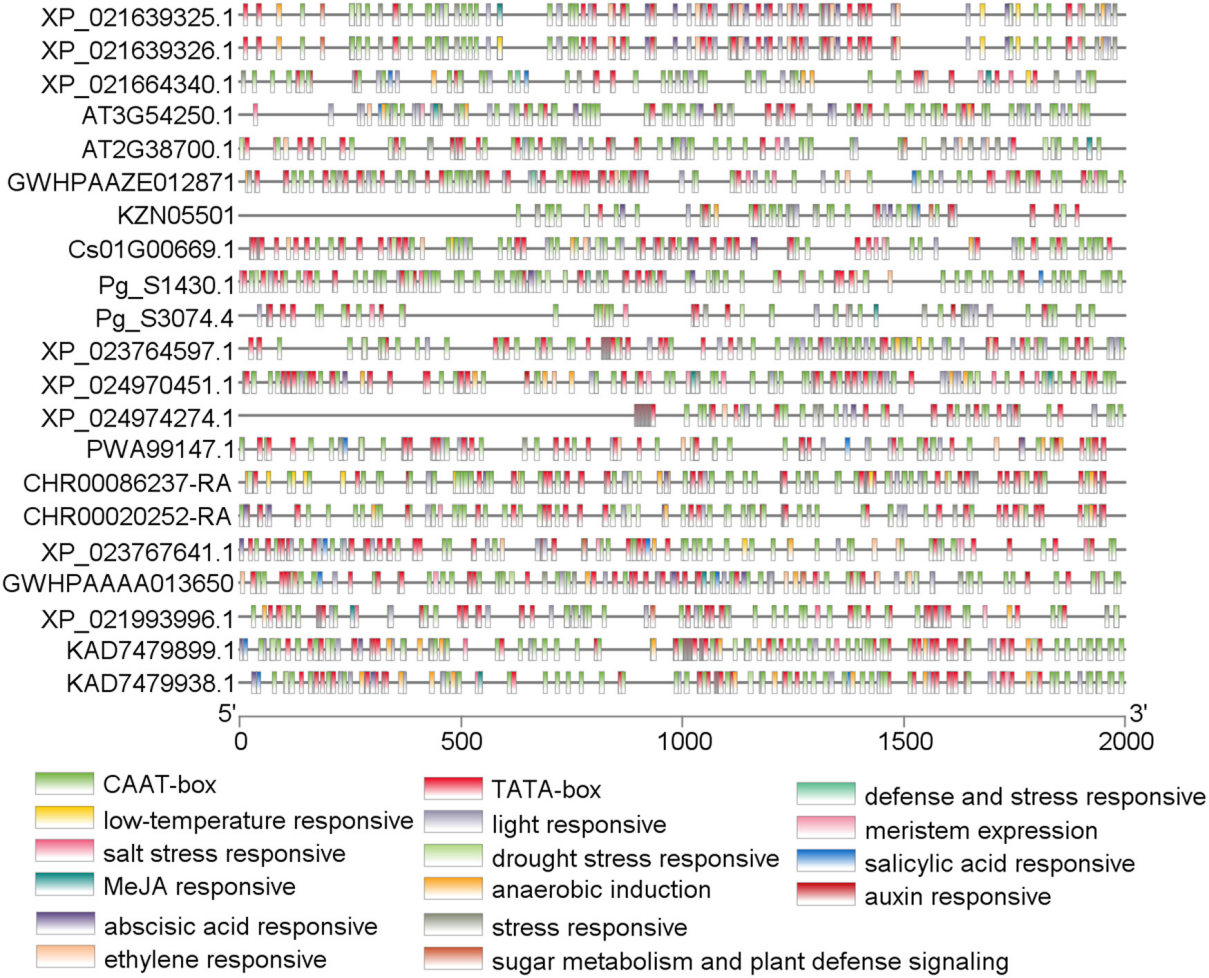
Prediction results of *cis*-acting elements of *MPD* gene.

TBtools were used to obtain 2,000 bp upstream sequences of *MPD* initiation codons of 13 species. These sequences were used to predict *cis*-acting elements of the promoters using PlantCARE software (Figure 8). The findings showed that all sequences had core cis-acting CAAT box and TATA box. Moreover, 24 components including G-box, MRE, Myb-binding site, and Box 4 were identified in the sequences. Other elements identified in most of the sequences included abscisic acid response elements WUN-motif and ABRE, salicylic acid homeostasis elements (TCA-element and SARE), auxin response elements (TGA-element and AuxRR-core), MeJA response elements (TGACG-motif and CCGTCA-motif), w-box of glucose metabolism and plant stress signal elements, CCGTCC-motif and CAT-box of meristem, MYB and DRE stress elements, low temperature response elements (as-1 and LTR), anaerobic induction element (ARE), drought stress elements (MBS and MYC), salt stress element (STRE), defense and stress response elements (JERE and TC-rich repeats), and ethylene response element (ERE). In addition, sequence specific elements were identified. These elements included; maize gliadin element O_2_-site in *P. ginseng* (Pg_S3074.4 and Pg_S1430.1) and *H. brasiliensis* (XP_021639325.1, XP_021664340.1, and XP_021639326.1); endosperm expression element GCN4-motif in *Coriandrum sativum* (Cs01G00669.1), *Cynara cardunculus* var. *scolymus* (XP_024974274.1), and *Arabidopsis thaliana* (AT3G54250.1); gibberellin response element P-box, TATC-box, and GARE-motif in *H. brasiliensis* (XP_021639325.1, XP_021664340.1, and XP_021639326.1), *Arabidopsis thaliana* (AT3G54250.1), *Cynara cardunculus* var. *scolymus* (XP_024974274.1), *Lactuca sativa* (XP_023764597.1), *Helianthus annuus* (XP_021993996.1), and *Mikania micrantha* (KAD7479899.1 and KAD7479938.1); flavonoid biosynthetic elements MBSI in *Lactuca sativa* (XP_023767641.1) and *Chrysanthemum nankingense* (CHR00086237-RA); circadian regulatory elements in *Artemisia annua* (PWA99147.1) and *Arabidopsis thaliana* (AT2G38700.1); mesophyll cell differentiation element HD-zip1 in *P. ginseng* (Pg_S3074.4 and Pg_S1430.1) and *Taraxacum kok-saghyz* (GWHPAAAA013650); hypoxia specific inducible element GC-motif in *Mikania micrantha* (KAD7479938.1); and specific regulatory element RY-element in *H. brasiliensis* (XP_021664340.1). Structure prediction results for *MPD* gene are shown in Figure S2.

## 4 Discussion

In the current study, 21 *MPD* gene sequences of 11 species of *Campanulids* were searched and retrieved by local BLAST. Analysis of physicochemical properties showed that *MPD* proteins are hydrophilic proteins without a transmembrane region. The average length of *MPD* proteins was approximately 420 amino acids with subtle differences in relative molecular weights and isoelectric points. Motif analysis further showed a total of 10 motifs in the 27 sequences. Most sequences showed high motif similarity. Adaptive evolution analysis of the *MPD* gene with *A. thaliana* and *H. brasiliensis* as outgroups showed that the *MPD* gene had a significant negative selection with high reliability in the evolution process. Moreover, the *MPD* gene on chromosome 2 of *D. carota* was collinear with that on chromosome 1 of *C. sativum*. This finding indicates a close genetic relationship between the two species. This finding was consistent with findings from phylogenetic analysis. Analysis of cis-acting elements of each *MPD* gene sequence showed presence of core elements (CAAT-box and TATA-box) in the upstream of each *MPD* sequence and several light response elements such as G-box. In addition, several cis-acting elements such as abscisic acid response elements, salicylic acid homeostasis elements, and auxin response elements were observed.

The findings showed that selection pressure of each branch of the *MPD* gene was different owing to the protein’s physical and chemical properties as well as results from adaptive evolution analysis, motif analysis, and cis-acting element analysis. However, all sequences had an influence of net selection i.e., *ω* < 1 which indicated that the *MPD* gene may have significantly negative pressure in the evolution process. The structure of selective action is extremely conservative [19]. Notably, damarenediol synthetase, an essential enzyme in ginsenoside biosynthesis pathway is highly conserved. *P. ginseng* and *P. notoginseng* proteins differ only by 8 amino acids. However, their similarity is approximately 98% [23]. This is attributed to the conserved nature of the enzyme during evolution process to maintain stability of the structure and function of the enzyme as it is an important enzyme in synthesis of triterpenoids. Moreover, this can be attributed to less variations or genes involved in analysis and comparison as a consequence of having undergone adaptive evolution in an earlier period. Therefore, the evolutionary signals may have been submerged by the general medium-sized or purified selection [24].

The findings showed that the residues 150 to 350 of *MPD* protein were highly conserved, whereas NCBI-CDD identification showed that the conserved domain (domain accession: PLN02407) of *MPD* protein comprises residue 10 to 350. MEC analysis showed that there was no dark purple site after the residue 350 of *MPD* protein. This finding indicates that there was no negative selection site after this residue, implying that the first 350 amino acids of *MPD* protein, mainly amino acids at positions 150–350, were highly conserved. Further, prediction of the three-dimensional structure shows that this conserved domain may be responsible for binding to 2 × DP6: (3*R*)-3-hydroxy-5-{[(*R*)-hydroxy (phophotonooxy) phophoryl] oxy}-3-methylpentanoic acid. Therefore, it forms the active site of *MPD* protein.

Analysis of cis-acting elements showed presence of several elements involved in light responses. Studies report that expression of the *MPD* gene may be affected by light. TGACG-motif and the CCGTCA-motif were present in most *MPD* gene promoter sequences. Previous studies report two types of methyl jasmonate (MeJA) response elements. Jin et al., 2017 reported that treatment of *E. senticosus* with appropriate concentration of MeJA increased the saponin yield. This indicates a positive correlation between concentration of MeJA and intensity of *MPD* expression [25]. Moreover, Shi et al., 2012 reported that a concentration of MeJA at 150 μmol/L increased activity of the *MPD* gene. However, activity of the *MPD* gene in *Ganoderma lucidum* was inhibited by 100 μmol/L of MeJA. These findings indicate that different concentrations of MeJA negatively affects expression of the *MPD* gene in *Ganoderma lucidum*. Studies on the *MPD* gene of *H. brasiliensis* report that MeJA modulates expression intensity of *MPD* [26]. The current study shows that *MPD* gene was expressed in all parts of *H. brasiliensis*. The cis-acting elements of the *MPD* gene in *H. brasiliensis* were zein, seed specific regulatory elements, and endosperm expression elements. This finding was consistent with findings from previous studies which reported that in addition, expression of *MPD* in female flowers was higher compared with the levels in other parts. This result was similar to findings by Xing ZB et al., 2013 that the expression level of *MPD* in female *E. senticosus* plants was significantly higher compared with that of male plants [27]. These findings indicate that the expression level of *MPD* depends on the location in male or female parts. Tolerance of female plants to environmental stress is lower compared with that of male plants [28]. In a damage study using *H. brasiliensis*, expression of *MPD* increased after the plants were damaged by knocking. Several elements implicated in stress response were detected in the cis-acting elements *H. brasiliensis* after damage [26]. These findings imply that the stress elements in the promoter sequence of the *MPD* gene modulate expression process of *MPD*.

## 5 Conclusion

The findings from the current study show that *MPD* gene is highly conserved. This property is a possible characteristic of all essential enzymes in biosynthesis pathways of medicinal plants. *MPD* gene is significantly affected by environmental factors which subsequently modulate its expression. The findings of the current study provide key information and a reference for follow-up studies on the *MPD* gene and essential enzymes in other medicinal plants.
